# Machilin D Promotes Apoptosis and Autophagy, and Inhibits Necroptosis in Human Oral Squamous Cell Carcinoma Cells

**DOI:** 10.3390/ijms24054576

**Published:** 2023-02-26

**Authors:** Hyung-Mun Yun, Yoon-Ju Kwon, Eonmi Kim, Hea-Jong Chung, Kyung-Ran Park

**Affiliations:** 1 Department of Oral and Maxillofacial Pathology, School of Dentistry, Kyung Hee University, Seoul 02447, Republic of Korea; 2 National Development Institute for Korean Medicine, Gyeongsan 38540, Republic of Korea; 3 Gwangju Center, Korea Basic Science Institute (KBSI), Gwangju 61751, Republic of Korea

**Keywords:** apoptosis, autophagy, Machilin D, necroptosis, OSCC

## Abstract

Oral squamous cell carcinoma (OSCC) accounts for about 90% of all head and neck cancers, the prognosis is very poor, and there are no effective targeted therapies. Herein, we isolated Machilin D (Mach), a lignin, from the roots of *Saururus chinensis* (*S. chinensis*) and assessed its inhibitory effects on OSCC. Herein, Mach had significant cytotoxicity against human OSCC cells and showed inhibitory effects against cell adhesion, migration, and invasion by inhibiting adhesion molecules, including the FAK/Src pathway. Mach suppressed the PI3K/AKT/mTOR/p70S6K pathway and MAPKs, leading to apoptotic cell death. We investigated other modes of programmed cell death in these cells and found that Mach increased LC3I/II and Beclin1 and decreased p62, leading to autophagosomes, and suppressed the necroptosis-regulatory proteins RIP1 and MLKL. Our findings provide evidence that the inhibitory effects of Mach against human YD-10B OSCC cells are related to the promotion of apoptosis and autophagy and inhibition of necroptosis and are mediated via focal adhesion molecules.

## 1. Introduction

*Saururus chinensis* is a perennial herb whose roots have been used for treating edema, jaundice, gonorrhea, and inflammation in Korea and other Asian countries [1]. Many compounds have been isolated from *Saururus* species, including lignans, aristolactams, flavonoids, anthraquinones, and furanoditerpene [2]. *S. chinensis* extracts and isolated compounds have various pharmacological and biochemical effects, i.e., antioxidant, anti-inflammatory, and anti-tumor activities [1,3,4]. Recently, the effects of compounds isolated from *S. chinensis* on several cancer cells, including breast cancer, gastric cancer, and hepatocellular carcinoma cells have been investigated. The compounds isolated from *S. chinensis* showed anti-cancer effects by targeting of the AMPK-mTOR pathway in hepatocellular carcinoma, by inhibiting TGF-β-induced epithelial-mesenchymal transition and metastasis in gastric cancer cells, and also preventing IL-6 and IL-8 signaling in breast cancer stem cells [5,6,7]. Therefore, *S. chinensis* is considered a valuable resource of bioactive compounds aimed at treating various diseases. Machilin D (Mach), a lignin from *S. chinensis* roots, exhibited antioxidant activities, macrophage-mediated low-density lipoprotein oxidation [3,8], and significant cardiovascular effects, including vasorelaxant and negative inotropic activities [9]. Recently, it was reported that Mach inhibits the NF-κB pathway and breast cancer stem cell growth [7]. However, there are no reports on the effect of Mach on human oral squamous cell carcinoma (OSCC).

OSCC is the most common malignancy of the head and neck, and it frequently metastasizes to lymph nodes [10,11]. It is the sixth leading cause of cancer-related death globally. More than 50% of the OSCC patients die from the disease [12,13]. Human papilloma virus infection is associated with OSCC pathogenesis, and drinking and smoking are well-known OSCC risk factors [14,15,16,17]. Although OSCC etiology, development, and progression are well elucidated, OSCC is still a life-threatening condition, owing to aggressive features, including metastasis and recurrence [10,18].

In the present study, we purified Mach from *S. chinensis* root extract (>95% purity) and investigated its effects on the proliferation, adhesion, migration, invasion, apoptosis, autophagy, and necroptosis of OSCC cells.

## 2. Results

### 2.1. Isolation and Identification of Mach

**Figure 1:**
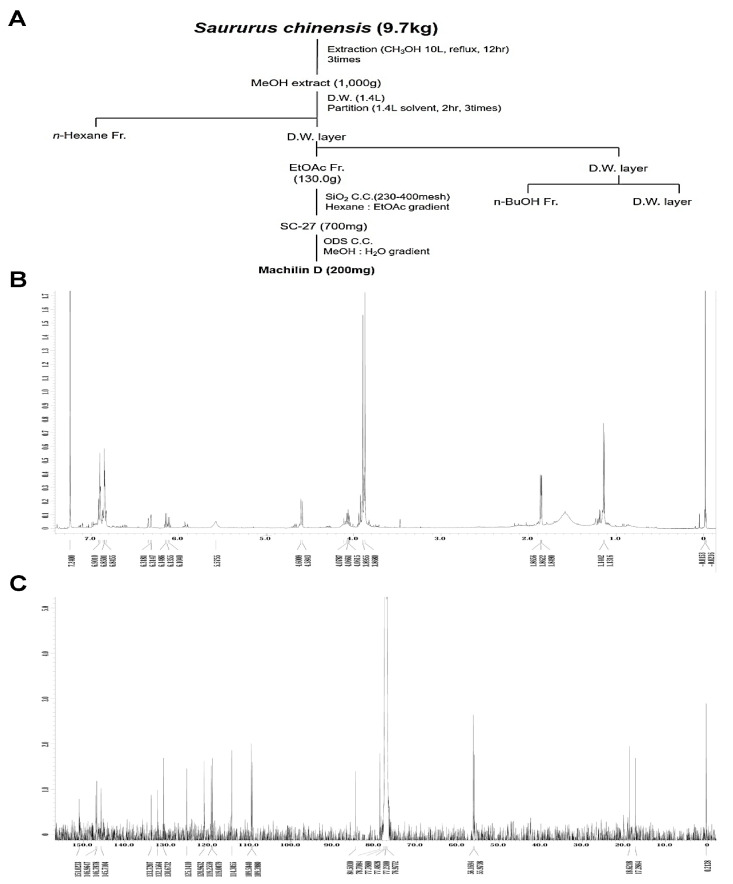

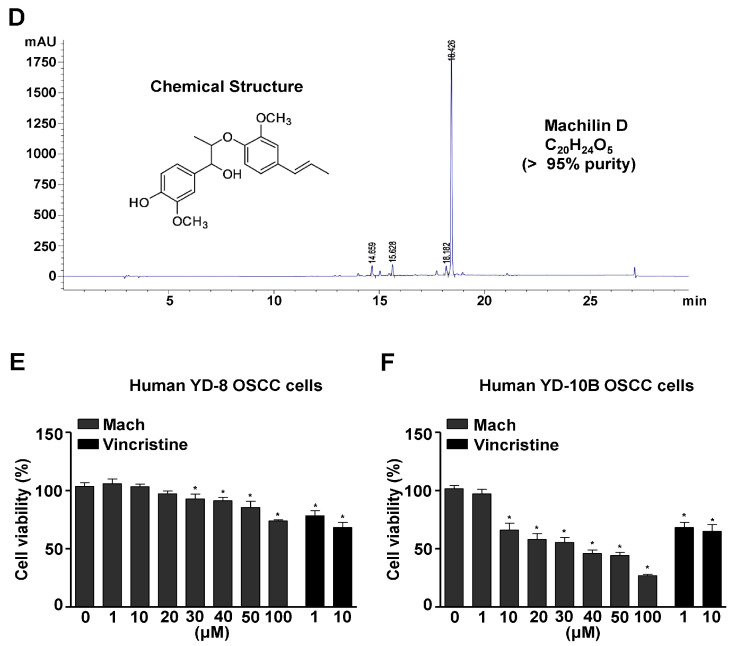
Isolation of Machilin D (Mach) from the roots of *Saururus chinensis* and its cytotoxic effects on OSCC cells. (**A**) Schematic of the method used to isolate and purify Mach from the roots of *S. chinensis*. (**B**,**C**) The ^1^H-NMR (500 MHz, CDCl_3_) (**B**) and ^13^C-NMR (125 MHz, CDCl_3_) (**C**) spectra of Mach. (**D**) HPLC analysis of purified Mach. The chemical structure, purity, and molecular formula of Mach are shown (inset). (**E**,**F**) Cell viability (%) was measured using the MTT assay after treatment with Mach (1, 10, 20, 30, 40, 50, and 100 μM) or Vincristine (1 and 10 μM) for 24 h in human YD-8 OSCC cells (**E**) and YD-10B OSCC cells (**F**). Data are the mean ± SEM. Statistical significance was analyzed using Student’s unpaired *t*-test. Asterisks indicate statistical significance (* *p* < 0.05).

The method used to isolate Mach from *S. chinensis* roots (9.7 kg) is depicted in Figure 1A. ^1^H-NMR (500 MHz, CDCl_3_) assign data: *δ* 6.92~6.82 (6H, m, aromatic protons), 6.33 (1H, dd, *J* = 15.8, 1.7 Hz, H-7′), 6.13 (1H, m, H-8′), 5.58 (1H, brs, -OH), 4.59 (1H, d, *J* = 8.3 Hz, H-7), 4.08 (1H, m, H-8), 3.90 (3H, s, O-CH_3_), 3.87 (3H, s, O-CH_3_), 1.86 (3H, dd, *J* = 6.6, 1.7 Hz, H-9′), 1.14 (3H, d, *J* = 6.3 Hz, H-9) (Figure 1B and Supplementary Figure S1A). ^13^C-NMR (125 MHz, CDCl_3_) assign data: *δ* 151.0 (C-3′), 147.0 (C-3), 146.8 (C-4′), 145.7 (C-4), 133.7 (C-1′), 132.2 (C-1), 130.7 (C-7′), 125.1 (C-8′), 121.0 (C-6), 119.2 (C-6′), 119.0 (C-5′), 114.3 (C-5), 109.6 (C-2′), 109.4 (C-2), 84.5 (C-8), 78.7 (C-7), 56.2 (O-CH_3_), 56.0 (O-CH_3_), 18.6 (C-9′), 17.3 (C-9) (Figure 1C and Supplementary Figure S1B). The HPLC chromatogram and chemical structure (inset, Figure 1D) of Mach (a colorless oil, molecular formula: C_20_H_24_O_5_, purity: >95%) are shown in Figure 1D. 

### 2.2. Mach Is Cytotoxic to Human OSCC Cells

To examine the cytotoxicity of Mach, wild-type p53 YD-8 and mutant p53 YD-10B OSCC cells were treated with various concentrations of Mach (0, 1, 10, 20, 30, 40, 50, and 100 μM) for 24 h. Mach significantly reduced the viability (%) of both YD-8 OSCC cells (1 μM: 95.27 ± 5.82, 10 μM: 93.97 ± 3.06, 20 μM: 84.43 ± 0.75, 30 μM: 73.93 ± 2.08, 40 μM: 62.27 ± 6.61, 50 μM: 62.50 ± 1.89, and 100 μM: 53.57 ± 3.38) and YD-10B OSCC cells (1 μM: 91.28 ± 4.49, 10 μM: 77.56 ± 5.31, 20 μM: 71.10 ± 2.78, 30 μM: 45.82 ± 1.33, 40 μM: 39.17 ± 3.57, 50 μM: 37.27 ± 3.59, and 100 μM: 21.22 ± 0.23) (Figure 1E,F), indicating that mutant p53 YD-10B OSCC cells were more sensitive to Mach than wild-type p53 YD-8 cells. Vincristine is widely used to treat various malignancies as a chemotherapeutic agent, it was used as a positive control because Vincristine is well known to block cell growth and mitosis by binding to microtubular proteins of the mitotic spindle and preventing the polymerization of tubulin to form microtubules [19,20]. When comparing the inhibitory effects of Mach and Vincristine, Mach was more effective than Vincristine at concentrations above 10 μM. Based on these data, 10, 30, and 50 μM Mach was used to treat YD-10B cells in subsequent experiments.

### 2.3. Mach Inhibits Cell Adhesion to Extracellular Matrix, Migration, Invasion of Human OSCC Cells, and FAK/Src Signaling

**Figure 2:**
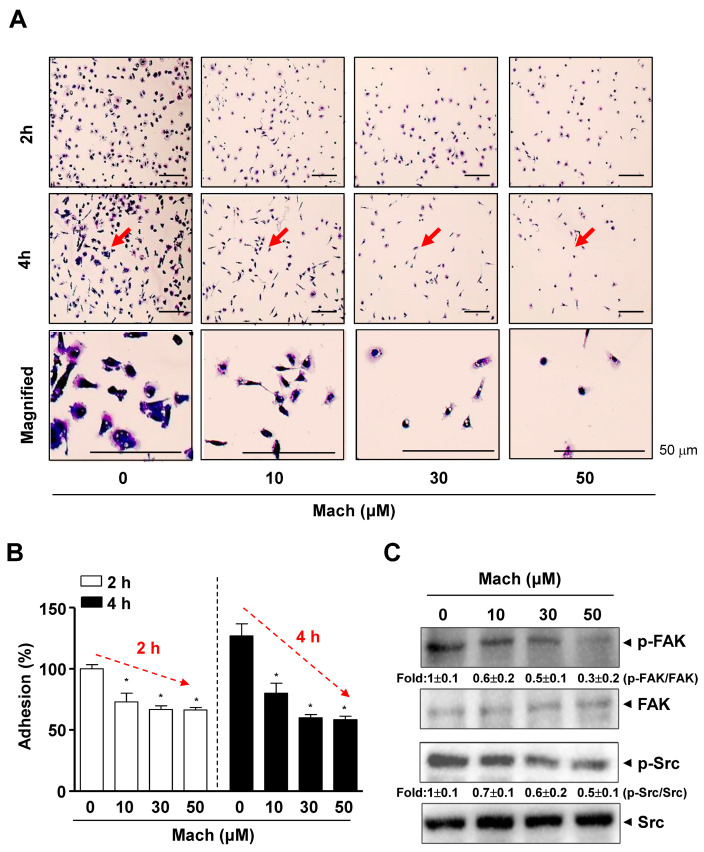
Effects of Mach on cell adhesion and Fyn/Src signaling in human YD-10B OSCC cells. (**A**,**B**) Cell adhesion on ECM-coated plates was observed after treatment with Mach for 2 and 4 h using a light microscope. The magnified regions are indicated by arrows. Scale bar: 50 μm (**A**). Relative adhesion is shown as a bar graph (**B**). (**C**) Western blotting of phospho-FAK (p-FAK), FAK, phospho-Src (p-Src), and Src levels. Data are the mean ± SEM. Statistical significance was analyzed using Student’s unpaired *t*-test. Asterisks indicate statistical significance (* *p* < 0.05).

As the prognosis of OSCC, especially metastatic OSCC, is very poor, due to its aggressive features, we examined whether Mach has anti-metastatic activities. We conducted a cell adhesion assay to assess the attachment of cells to the extracellular matrix (ECM), which is related to metastasis and found that the adhesion of YD-10B OSCC cells to ECM was significantly attenuated by Mach in a time- and dose-dependent manner (compared to untreated control) (Figure 2A,B). Magnified images showed the altered morphology of Mach-treated OSCC cells, even cells that were attached to the ECM (Figure 2A). To examine the mechanism underlying the effects of Mach on OSCC cell adhesion, we focused on focal adhesion kinase (FAK)/Src (a proto-oncogene tyrosine-protein kinase) as FAK/Src signaling promotes cell adhesion, migration, and invasion, and these molecules are central in therapeutic approaches against OSCC [13]. We investigated the effects of Mach on these molecules using Western blotting, which showed that Mach inhibited FAK and Src phosphorylation with mild effects at 10 and 30 μM and clear effects at 50 μM (Figure 2C).

**Figure 3:**
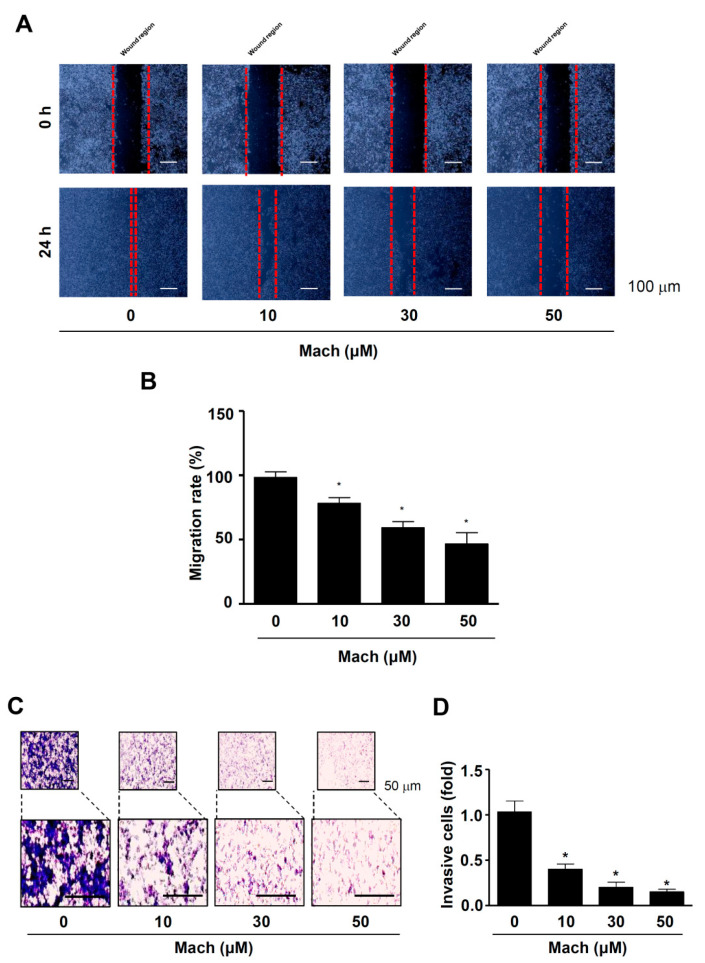
Effects of Mach on cell migration and invasion of human YD-10B OSCC cells. (**A**,**B**) Wound healing assay to assess cell migration. Migration was observed using a light microscope. Scale bar: 100 μm (**A**). Relative migration (%) is shown as a bar graph (**B**). (**C**,**D**) Boyden chamber assay to assess cell invasion. Invasive cells were imaged using a light microscope. Scale bar: 50 μm (**C**). Relative invasion is shown as a bar graph (**D**). Data are the mean ± SEM. Statistical significance was analyzed using Student’s unpaired *t*-test. Asterisks indicate statistical significance (* *p* < 0.05).

We also performed a wound healing assay to evaluate cell migration and found that Mach significantly suppressed the migration of YD-10B cells into the wound areas in a dose-dependent manner (compared to untreated control cells) (Figure 3A,B). Next, we performed a Boyden chamber assay to monitor cell invasion through ECM, which showed that Mach significantly attenuated the transmigration of YD-10B OSCC cells across a Matrigel-coated membrane (10 μM: 0.61 ± 0.13, 30 μM: 0.32 ± 0.11, 50 μM: 0.07 ± 0.03) (Figure 3C,D).

### 2.4. Mach Inhibits the PI3K/AKT/mTOR/p70S6K Pathway in OSCC Cells

**Figure 4:**
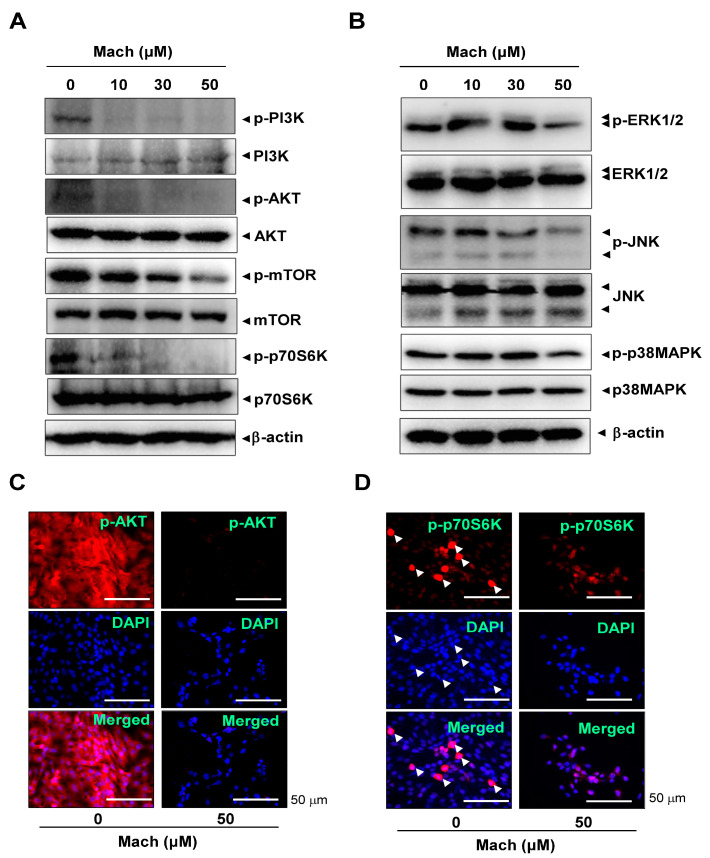
Effects of Mach on the PI3K/AKT/mTOR/p70S6K and MAPKs pathways in human YD-10B OSCC cells. (**A**) Western blotting of phospho-PI3K (p-PI3K), PI3K, phospho-AKT (p-AKT), AKT, phospho-mTOR (p-mTOR), mTOR, phospho-p70S6K (p-p70S6K), p70S6K, and -actin levels. (**B**) Western blotting of phospho-ERK1/2 (p-ERK1/2), ERK1/2, phospho-JNK (p-JNK), JNK, phospho-p38 (p-p38) MAPK, p38 MAPK, and β-actin levels. (**C**,**D**) Immunofluorescence assay to assess phosphorylation levels of AKT (**C**) and p70S6K (**D**) in YD-10B OSCC cells. Images were captured using a microscope. Nuclei were stained with DAPI (blue). Scale bar: 50 μm. Data are representative of the results from three separate experiments. Statistical significance was analyzed using Student’s unpaired *t*-test.

The PI3K/protein kinase B (AKT)/mammalian target of rapamycin (mTOR)/p70S6K pathway is frequently amplified in OSCC [21,22]. We investigated the effects of Mach on the PI3K/AKT/mTOR/p70S6K pathway using Western blotting and found that Mach inhibited the phosphorylation of PI3K and AKT and the downstream targets mTOR and p70S6K in YD-10B cells (Figure 4A). Mach also inhibited the activities of the mitogen-activated protein kinases (MAPKs) ERK1/2 and JNK (Figure 4B). We further observed AKT and p70S6K phosphorylation using a fluorescence microscope, which showed that Mach suppressed the PI3K/AKT/mTOR/p70S6K pathway in YD-10B cells (Figure 4C,D). Notably, observation of p-p70S6K- and DAPI-stained cells showed that Mach attenuated the division of OSCC cells (compared to control) (arrowheads in Figure 4D).

### 2.5. Mach Promotes Caspase-Dependent Apoptosis and Reduces Cell-Cycle Protein Levels in OSCC Cells

**Figure 5:**
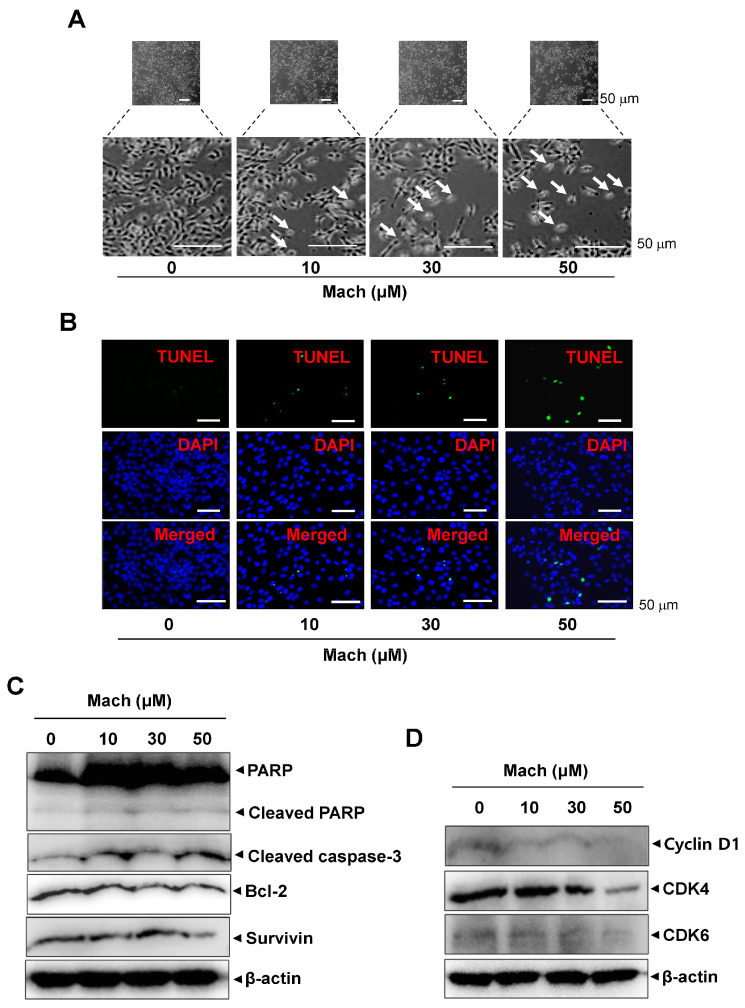
Effects of Mach on apoptosis in human YD-10B OSCC cells. (**A**) Apoptotic morphological changes were imaged using a light microscope. Scale bar: 100 μm. (**B**) Apoptosis was assessed using TUNEL (green) and DAPI (blue) staining. Images were obtained using a microscope. Scale bar: 50 μm. (**C**) Western blotting of PARP, cleaved PARP, cleaved caspase 3, Bcl-2, and Survivin levels. (**D**) Western blotting of cyclinD1, CDK4, and CDK6 levels. β-actin was included as a loading control. Data are representative of the results from three separate experiments. Statistical significance was analyzed using Student’s unpaired *t*-test.

To investigate whether programmed cell death is involved in the anti-cancer effects of Mach, we observed the morphological changes in Mach-treated YD-10B cells using a light microscope. Mach treatment induced a decrease in cell number and apoptotic changes in the cells indicated by arrows in magnified images, as evidenced by their small size, round shape, and weak attachment (Figure 5A). The apoptotic cell death was also further validated using a TUNEL assay, which showed that Mach increased the TUNEL-positive cells (compared to the control) (Figure 5B). Based on the Mach-induced apoptotic phenotypes, we further assessed apoptosis through the caspase cascade using Western blotting. Mach increased the cleavage of PARP and caspase 3 and down-regulated Bcl-2 and Survivin (Figure 5C). Moreover, Mach suppressed the expression of the cell division-promoting protein Cyclin D1, and CDK4 and CDK6 (Figure 5D). The results also showed the inhibition of CDK4 in high doses of Mach. Thus, the effects of Mach on CDK4 are less effective (Figure 5D).

### 2.6. Mach Induces Autophagy and Suppresses Necroptosis in OSCC Cells

**Figure 6:**
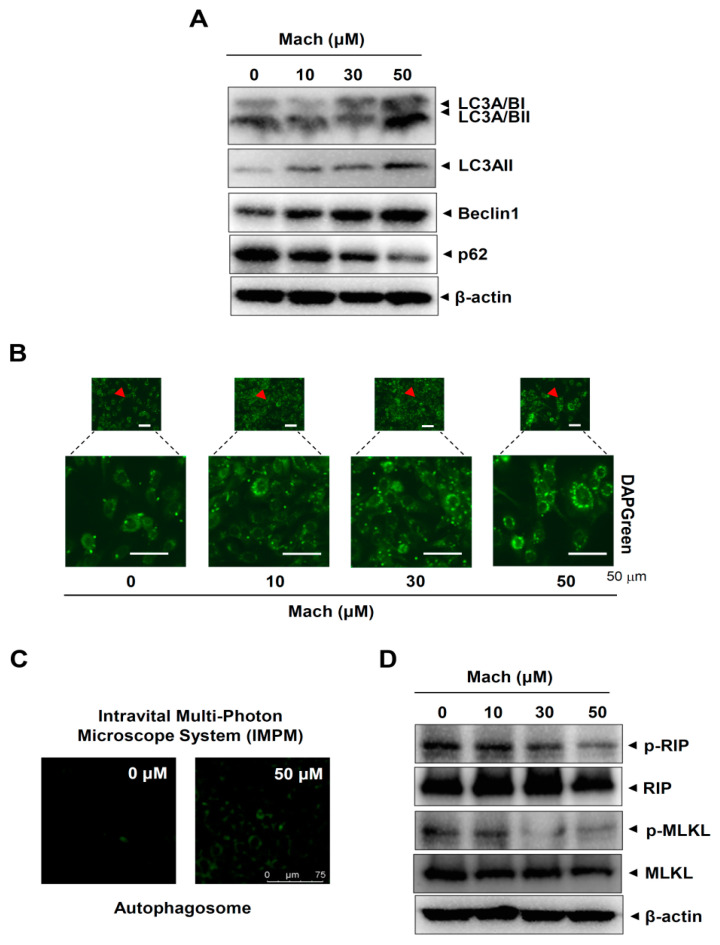
Effects of Mach on autophagy and necroptosis in human YD-10B OSCC cells. (**A**) Western blotting of LC3A/BI, LC3A/BII, LC3AII, Beclin1, p62, and β-actin levels. (**B**,**C**) DAPGreen-stained autophagosomes were imaged using a fluorescence microscope (arrowheads: magnified regions) (**B**) and an intravital multi-photon microscope system (IMPM) (**C**). (**D**) Western blotting of phospho-RIP (p-RIP), RIP, phospho-MLKL (p-MLKL), MLKL, and β-actin levels. Data are representative of the results from three separate experiments. Statistical significance was analyzed using Student’s unpaired *t*-test.

We next investigated whether Mach regulates two other types of programmed cell death, autophagy and necroptosis, in YD-10B cells. First, we analyzed autophagy markers using Western blotting and found that Mach up-regulated microtubule-associated protein light chain 3A/B (LC3A/B) and Beclin1 and down-regulated p62 (Figure 6A). We also observed autophagosome formation in these cells using a fluorescence microscope, which showed that Mach increased the formation of autophagic vacuoles (Figure 6B,C). Next, we analyzed two components of the necroptosis machinery, receptor-interacting serine/threonine-protein kinase (RIP) and mixed lineage kinase domain-like pseudokinase (MLKL), by Western blotting. The results showed that Mach decreased RIP and MLKL phosphorylation (Figure 6D). Overall, our findings suggest that Mach induces apoptosis and autophagy but blocks necrosis in OSCC.

## 3. Discussion

In the present study, we isolated the bioactive lignin Mach to >95% purity from *S. chinensis* roots and demonstrated its anti-OSCC effects. It was reported that p53 is mutated in approximately 70% of cases in OSCC, which is correlated with cell growth, invasion, and resistance to chemotherapeutic drugs, leading to the increased mitosis and abnormal genomic stability related with poor prognosis [23,24]. We demonstrated that Mach is shown to be more effective in mutant p53 YD-10B OSCC cells, indicating that Mach is a potentially targeted therapy in OSCC with these mutated genes.

Cell adhesion is related to the establishment of a new niche for the colonization of circulating tumor cells during metastasis [25]. FAK/Src signaling is involved in cell adhesion, migration, and invasion through various tumorigenesis- and progression-related pathways [13]. FAK expression in OSCC tissues significantly correlated with increased tumorigenesis and progression, supporting its role in OSCC [26]. Cell adhesion induces morphological changes through FAK/Src pathway activation, which promotes cell migration and invasion [27]. Here, we demonstrated that Mach suppresses adhesion of cells to the ECM and inactivates FAK/Src signaling in OSCC cells. The important processes in metastasis also include migration and invasion [28]. The invasion is a key step in the metastatic processes to penetrate and disseminate into the surrounding tissue from the primary tumor spreading, the cells invade across the basement membranes and migrate via the ECM [23,29]. In the present study, we showed that Mach suppresses OSCC cell migration in wound healing assays. Importantly, Mach more effectively inhibits OSCC cell invasion across the ECM, compared to OSCC cell migration. However, we found that the phosphorylation of FAK and Src is clearly inhibited at high concentrations of Mach. Although Mach has anti-tumoral effects, inhibiting the phosphorylation of Src and FAK is probably not the preferential molecular pathway. Thus, our results suggest that Mach exerts its anti-OSCC effects on cancer progression in part via FAK/Src signaling inhibition.

The PI3K/AKT/mTOR/p70S6K pathway increases cell proliferation and metastasis and decreases apoptosis and cell cycle arrest through various pathways in OSCC [30,31]. The pathway inhibitors were reported as promising candidates for the treatment of oral cancers [22]. Here, we found that Mach inhibits constitutively active PI3K, AKT, mTOR, and p70S6K. Induced 70S6K activation is critical for synthesizing proteins that drive the cell cycle and cell division [32,33]. We demonstrated that Mach suppresses the division of OSCC cells, which is consistent with p70S6K inactivation. Overexpression and activation of the PI3K/AKT/mTOR/p70S6K pathway has been observed in OSCC patients [34]. PI3K/AKT/mTOR/p70S6K pathway inactivation promoted OSCC cell apoptosis [35]. Here, we demonstrated that Mach induces apoptosis and inhibits rapid proliferation along with morphologic alterations, DNA fragmentation, cellular caspase events, and cell cycle regulatory proteins in OSCC. Apoptosis evasion in mutant cells contributes to tumorigenesis and progression [36]. According to recent studies, the AKT signaling inhibitor may be used as a treatment for patients with malignant tumors including OSCC [21]. Given that apoptosis is also involved in events that inhibit malignant tumor metastasis [37], our results suggest that Mach is a new bioactive compound that may block metastatic processes and induce apoptosis in OSCC by blocking the intracellular signaling pathway.

Autophagy and necroptosis are programmed cell death types, other than apoptosis. Autophagy is a self-degradative process involving lysosomal degradation that regulates cell survival by removing and recycling cellular components [38]. Research has shown that crosstalk between apoptosis and autophagy inhibits tumorigenesis and metastasis [39,40,41]. Here, we found that Mach up-regulated a marker of autophagy, LC3A/B, and the conversion of LC3 to LC3II in OSCC cells. Consequently, we found that Mach induced autophagosome formation in OSCC cells. In addition to its roles in cell survival and apoptosis, the AKT pathway also regulates autophagic induction. Inactivation of the AKT pathway induced autophagy and cell death [42,43]. Thus, Mach may enhance OSCC cell apoptosis by inducing autophagy.

Necroptosis is a caspase-independent, fail-safe type of programmed cell death [44]. It is triggered when a cell is injured under conditions in which apoptosis cannot be induced or when apoptosis-regulatory proteins are chemically and genetically blocked [45]. Apoptotic activation inhibits necroptosis through inactivation of RIP and MLKL, leading to oligomerization of MLKL and its translocation to the membrane [46,47]. Here, we also found that Mach suppresses these regulatory proteins (RIP and MLKL) in OSCC cells. Thus, our results suggest that Mach regulates OSCC cell survival by inducing apoptosis and autophagy and reducing necrosis.

In conclusion, OSCC is a life-threatening disease, and patients with OSCC often experience a decline in their quality of life. Its high mortality rate is due its aggressive phenotypes, including rapid growth, frequent metastasis, and chemotherapeutic resistance [10,18]. Despite recent advances in diagnosis and treatment, there are important limitations to overcome, especially through the identification of novel bioactive compounds. Currently, several clinical studies of OSCC are targeting intracellular signaling molecules, and epidermal growth factor receptor inhibitors are commercially available for OSCC [13,21,22]. Our study is the first to provide evidence that Mach blocks OSCC cell adhesion, migration, invasion, proliferation, and survival by inhibiting the intracellular signaling pathway. Our data also demonstrated that Mach much more strongly inhibits invasion than motility, suggesting that it probably inhibits the ability of the cells to digest the ECM. Therefore, our findings indicates that Mach is a potential chemotherapeutic agent for OSCC.

## 4. Materials and Methods

### 4.1. General Procedures of Plant Material

Silica gel (70–230 mesh; Merck, Darmstadt, Germany) and reversed-phase (Lichroprep RP-18, 40–63 μm; Merck, Darmstadt, Germany) column chromatography was conducted. ^1^H-NMR and ^13^C-NMR spectra were obtained on a Jeol ECX-500 spectrometer (JEOL Ltd., Akishima, Japan). High-performance liquid chromatography (HPLC) was performed using Agilent 1260 (Agilent Technologies, Santa Clara, CA, USA) with a C18 column (CAPCELL PAK C18, 5 μm, 4.6 mm × 250 mm). *S. chinensis* roots were purchased from a folk medicine market “Yakryong-si” in Daegu, Republic of Korea. A voucher specimen (P412) has been deposited in the Natural Products Bank, National Institute for Korean Medicine Development (NIKOM).

### 4.2. Extraction, Isolation, and Purity Analysis

*S. chinensis* roots (9.7 kg) were extracted three times with methanol (MeOH) by refluxing for 12 h, and the crude extracts were combined and concentrated in vacuo. The obtained residue (1.0 kg) was suspended in distilled water (1.4 L) and further partitioned with n-hexane, ethyl acetate (EtOAc), and n-butanol. The EtOAc-soluble fraction (130.0 g) was subjected to silica gel column chromatography eluted with a mixture of n-hexane-EtOAc (100:0 to 0:100, *v*/*v*) to obtain 39 fractions (SCE 1–39). Fraction SCE 27 (700 mg) was further purified by reverse-phase column chromatography eluted with a MeOH-H_2_O gradient (50:50 to 0:100, *v*/*v*) to obtain the active compound (200 mg). The active compound was identified as Machilin D by comparing its spectral data to the literature [48]. The conditions for purity analysis were as follows: column, Phenomenex Kinetex C18 (150 mm × 4.6 mm, 2.6 μm, 100 A); mobile phase, A, 0.1% trifluoroacetic acid in water, B, 0.1% trifluoroacetic acid in ACN; gradient, 2–100% B (0–20 min; gradient for 30 min); flow rate, 1 mL/min; detector, ELSD; injection volume, 3 μL (1 mg/mL). Purity was calculated as follows: (206.04486/217.43100) × 100% = 94.9001% (95%).

### 4.3. Cell Culture

The human OSCC cell lines YD-8 and YD-10B were obtained from the Korean Cell Line Bank (Seoul, Republic of Korea) and cultured in Dulbecco’s modified Eagle medium (WELGEME, Inc., Seoul, Republic of Korea) containing 10% fetal bovine serum and 1× Gibco^®^ antibiotic-antimycotic (Thermo Fisher Scientific, Waltham, MA, USA) at 37 °C in a humidified 5% CO_2_ atmosphere as described previously [49,50].

### 4.4. Cytotoxicity Analysis

YD-8 and YD-10B OSCC cells were seeded in 96-well plates and treated with Mach for 24 h. Cytotoxicity was assessed using the 3-[4,5-dimethylthiazol-2-yl]-2,5-diphenyltetrazolium bromide (MTT) assay (Sigma-Aldrich, St. Louis, MO, USA) as described previously [51]. Absorbance at 540 nm was detected using a Multiskan GO Microplate Spectrophotometer (Thermo Fisher Scientific, Waltham, MA, USA).

### 4.5. Cell Adhesion Assay

Cell adhesion was analyzed as described previously [52]. Mach-pretreated OSCC cells were seeded into Matrigel-coated (Corning Life Sciences, Tewksbury, MA, USA) 96-well plates and allowed to attach for 2 and 4 h. The plates were washed with 1 × PBS, fixed with 10% formalin for 10 min, and stained using 0.5% crystal violet for 5 min. The stained cells were evaluated under a light microscope. Adherent cells were quantified by solubilizing the stain in 100% DMSO (Sigma-Aldrich) and detecting the absorbance at 595 nm using a Multiskan GO Microplate Spectrophotometer (Thermo Fisher Scientific).

### 4.6. Cell Migration Assay

OSCC cells were seeded in 6-well plates and scratched using a 200 μL pipette tip. Then, the scratched monolayers were incubated with or without Mach for 24 h. Images of cells migrating into the wound area were captured using a light microscope as described previously [53].

### 4.7. Cell Invasion Assay

A Boyden chamber assay was performed to investigate cell invasion as described previously [53]. Nucleopore filters were coated with Matrigel (Corning Life Sciences), and Mach-pretreated OSCC cells were seeded and allowed to infiltrate for 4 h. Images of invading cells were captured using a light microscope as described previously [53].

### 4.8. Apoptosis Assay

Apoptotic DNA fragmentation was monitored by terminal deoxynucleotidyl transferase-mediated FITC–dUDP nick-end labeling (TUNEL) assay using the in situ Cell Death Detection Kit (Roche Diagnostics GmbH, Mannheim, Germany) as described previously [53]. Briefly, OSCC cells were incubated with TUNEL reaction mixture for 1 h at 37 °C in the dark and then stained with 4′,6′-diamidino-2-phenylindole dihydrochloride (DAPI, Sigma-Aldrich) for 5 min. Images of apoptotic cells with DNA fragmentation were captured using a fluorescence microscope.

### 4.9. Western Blotting

Western blotting was performed as described previously [54]. Briefly, after measuring protein concentration using the Bradford reagent (Bio-Rad, Hercules, CA, USA), equal protein (20 μg) was separated through SDS-PAGE and transferred onto PVDF membranes. The membranes were blocked with 5% skimmed milk at room temperature for 1 h, probed overnight with primary antibodies at 4 °C, washed three times with 1× TBST, and then incubated with secondary antibodies (1:10,000; Jackson ImmunoResearch, West Grove, PA, USA) at room temperature for 1 h. After washing three times, the membranes were incubated with enhanced chemiluminescence reagent (Millipore, Bedford, MA, USA); images were captured using the ProteinSimple detection system (ProteinSimple, Santa Clara, CA, USA).

The following antibodies were used: p-PI3K (1:1000, #4228), PI3K (1:1000, #4257), p-AKT (1:1000, #4060), AKT (1:1000, #4691), p-mTOR (1:1000, #2974), mTOR (1:1000, #2983), p-p70S6K (1:1000, #9204), p70S6K (1:1000, #2708), p-ERK1/2 (1:2000, #9101), ERK1/2 (1:2000, #9102), p-p38MAPK (1:1000, #9211S), p38MAPK (1:1000, #9212), p-JNK (1:500, #9251), JNK (1:1000, #9252), FAK (1:1000, #3285), p-FAK (1:1000, #3283), Src (1:1000, #2109), p-Src (1:1000, #6943), PARP (1:1000, #9542), cleaved-caspase-3 (1:1000, #9661), Bcl-2 (1:1000, #15071), Survivin (1:1000, #2808), LC3A (1:1000, #4599), LC3A/B (1:1000, #12741), Beclin1 (1:1000, #3495). p62 (1:1000, #5114), p-RIP (1:1000, #65746), RIP (1:1000, #3493), p-MLKL (1:1000, #91689), and MLKL (1:1000, #14993) all from Cell Signaling Technology (Beverly, MA, USA); β-actin (C4, 1:1000, #sc-47778), CyclinD1 (1:1000, #sc-20044), CDK4 (1:1000, #sc-23896), and CDK6 (1:1000, #sc-7961) all from Santa Cruz Biotechnology (Santa Cruz, CA, USA).

### 4.10. Immunofluorescence

Immunofluorescence was performed as described previously [53]. Briefly, OSCC cells were blocked with 3% BSA at room temperature for 1 h and then probed overnight with primary antibodies at 4 °C. After washing three times, the cells were probed with secondary antibodies (Invitrogen, Carlsbad, CA, USA) for 2 h at room temperature. After washing three times, the cells were stained with DAPI (Sigma-Aldrich) for 5 min. Immunofluorescence images were captured using a fluorescence and laser scanning confocal microscope (Leica TCS SP5 AOBS/Tandem) at the Gwangju Center of Korea Basic Science Institute (KBSI).

### 4.11. Autophagy Assay

An autophagy assay was performed as described previously [55] using the DAPGreen Autophagy Detection Kit (Dojindo, Japan) to detect autophagosome formation according to the manufacturer’s instructions. Briefly, OSCC cells were probed with DAPGreen (0.1 μM) for 30 min, washed twice with culture medium, and treated with Mach for 6 h. Images of autophagosomes were captured using a fluorescence microscope and an intravital multi-photon microscope system (IMPM) at Gwangju Center, Korea Basic Science Institute (KBSI).

### 4.12. Statistical Analysis

Data were analyzed using GraphPad Prism version 5 (GraphPad Prism, Inc., San Diego, CA, USA). All values are reported as mean ± standard error of the mean (SEM). Significance was analyzed using Student’s unpaired *t*-test; *p* < 0.05 was considered significant.

## Data Availability

The data generated during the current study are available from the corresponding author on reasonable request.

## Supplementary Materials

The following supporting information can be downloaded at: https://www.mdpi.com/article/10.3390/ijms24054576/s1.

## Abbreviations

AKT	Protein kinase B
FAK	Focal adhesion kinase
Mach	Machilin D
mTOR	Mammalian target of rapamycin
MTT	3-[4,5-dimethylthiazol-2-yl]-2,5-diphenyltetrazolium bromide (MTT)
OSCC	Oral squamous cell carcinoma
