# Supplementary material for: Machilin D Promotes Apoptosis and Autophagy, and Inhibits Necroptosis in Human Oral Squamous Cell Carcinoma Cells
Source: Int J Mol Sci. 2023 Feb 26;24(5):4576. doi: 10.3390/ijms24054576 (PMC10002565; doi:10.3390/ijms24054576)

Supplementary Figure Legend

- **Supplementary Figure S1. NMR of Machilin D (Mach) from the roots of Saururus chinensis. (A, B)** The ^{1}H-NMR (500 MHz, $CDCl_3$) (A) and ^{13}C-NMR (125 MHz, $CDCl_3$) (B) spectra of Mach.

Supplementary Figure S1

A

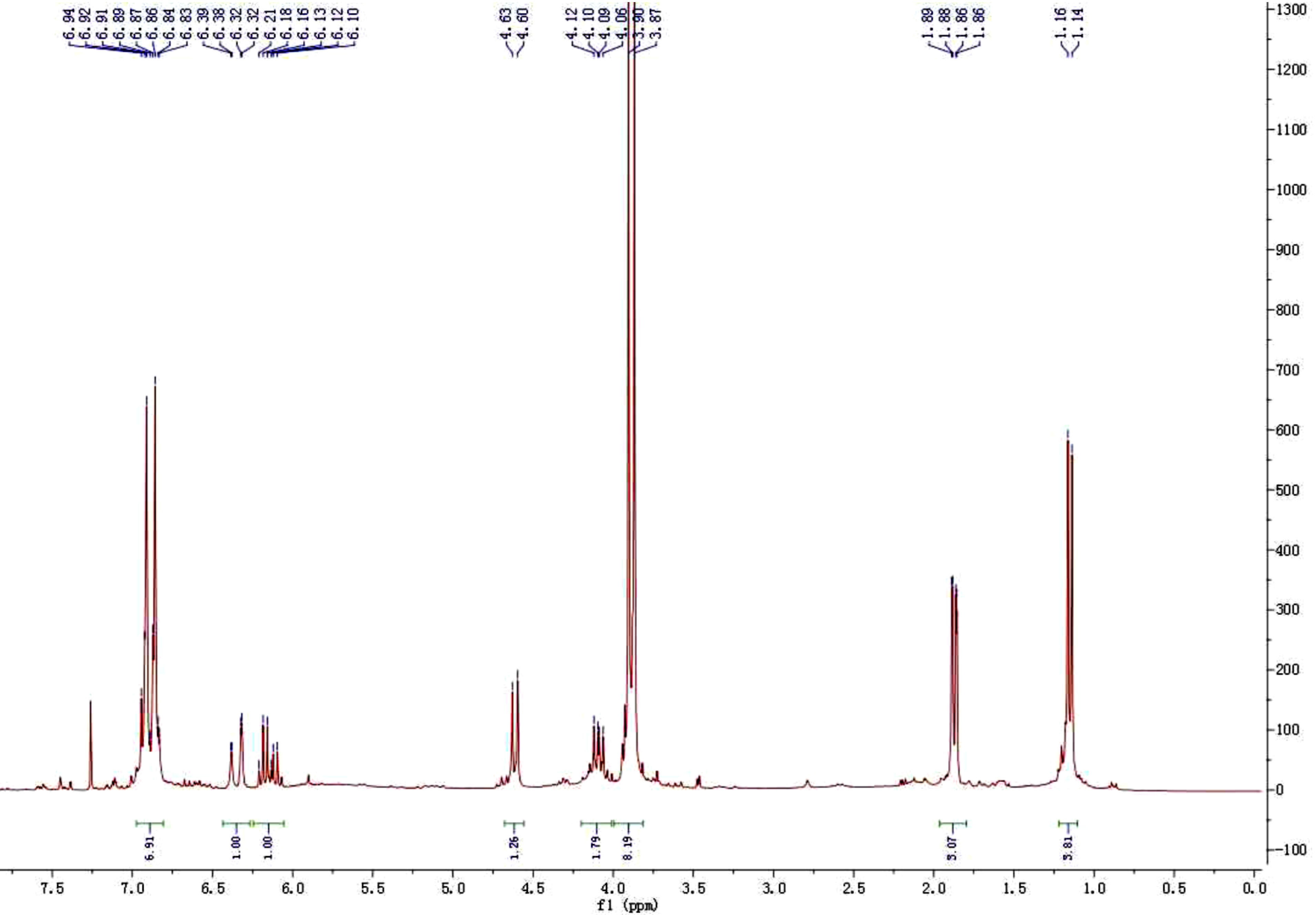

Supplementary Figure S1

B

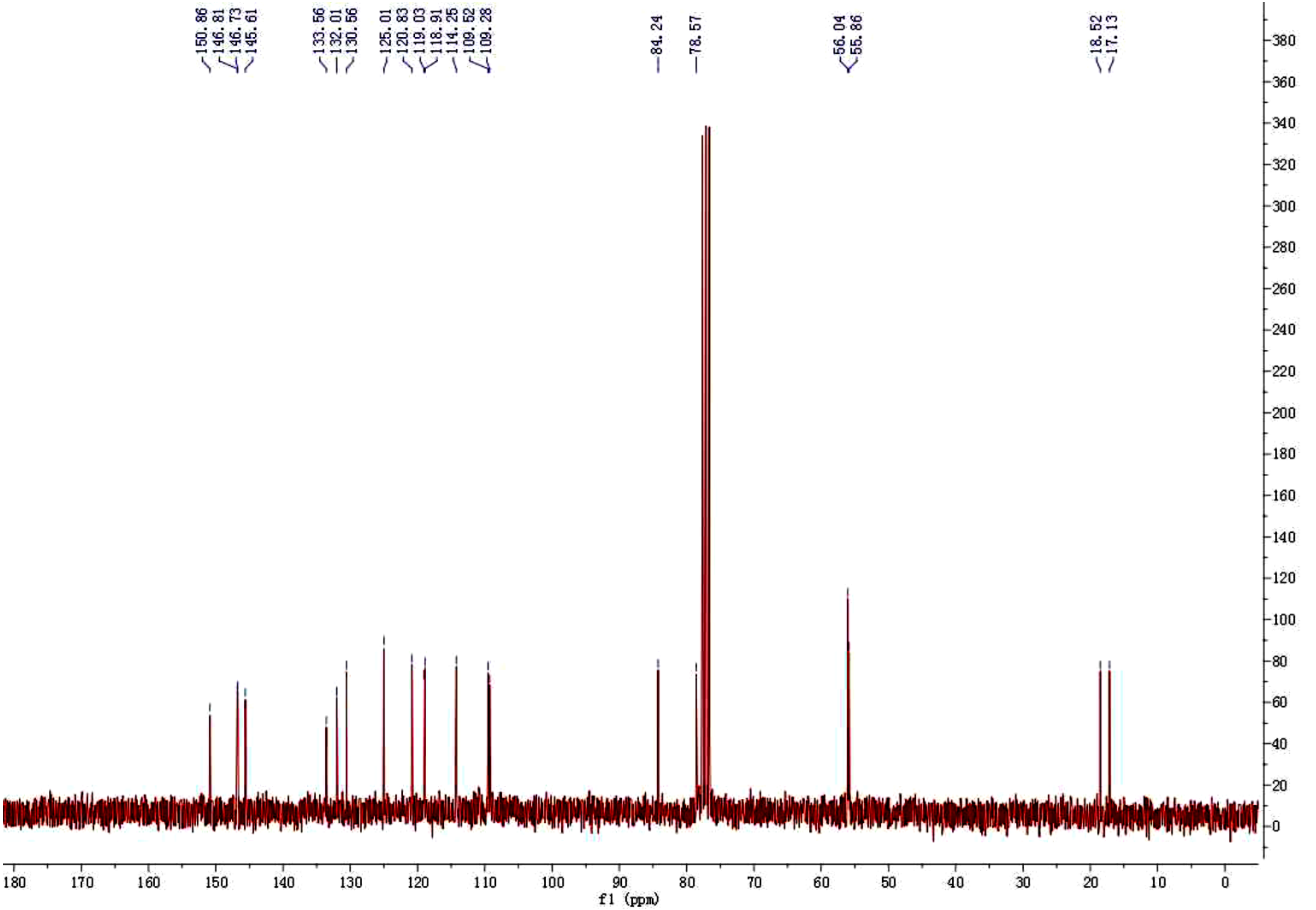

Supplement: Supplementary file 1 [file ijms-24-04576-s001.zip › ijms-2166118-supplementary.pdf]
